# Stem Cell Genetic Fidelity

**DOI:** 10.3389/fgene.2015.00051

**Published:** 2015-02-19

**Authors:** James L. Sherley

**Affiliations:** Asymmetrex, LLCBoston, MA, USA

**Keywords:** tissue stem cell, mutations, non-random segregation, immortal strands

This Frontiers Research Topic, “Stem Cell Genetic Fidelity,” is the product of an attempt to develop a comprehensive integrative treatment of the historical progression of ideas and research advances on the topic of biological mechanisms of gene mutation control in tissue stem cells, with human tissues as a primary interest. In retrospect, this undertaking began with a somewhat ambitious goal for achievement—beginning with its need to recruit authors diverse in both research discipline and generation. In this regard, the scientific characteristics of the final set of contributors are noteworthy. Although many more prospective authors were invited who work in primarily experimental research disciplines relevant to the topic, in the end these are underrepresented in the final volume. The ones for whom this volume's focus was an effective attractor constitute a select set of cell biologists and molecular biologists whose search for fundamental principles of tissue stem cell biology is driven as much by theoretical modeling approaches as by experimentation.

The deployment of modeling strategies by investigators of the biological principles of genetic fidelity in mammalian tissue stem cells is consistent with the challenges presented by this research. The low tissue fractions of stem cells and the low fractions of the mutations they incur combine to present investigation challenges that defy ideal quantitative analyses. Yet, at the same time, the intrinsic cascades of stem cell-based tissue cell lineages and molecular gene expression hierarchies synergistically amplify minute genetic-cellular deviations in ways that can profoundly influence life and health.

The final cast of contributors is well suited to achieving a particular goal of this Research Topic, which is to bring attention to remarkable advances in cell biology thought that flow from the theoretical genius of four giants in stem cell genetic fidelity research, Lark, Cairns, Potten, and Knudson. Though all applied observation and experimentation for discovering fundamental biological principles, they also employed elegant theoretical modeling as a fine tool for exploring and predicting the workings of tissue cells ahead of experimental approaches and confirmation. Lark, Cairns, and Potten are the most tightly woven into the fabric of ideas on the importance of tissue stem cell genetic fidelity in normal tissue function and carcinogenesis as a result to their shared contributions to the immortal strand hypothesis (Cairns, 1975). Subsequently, Knudson's two hit hypothesis gave wings to the idea that a key rate limiting factor for the emergence of tumors was two mutations, not only in the alleles of the same gene, but implicitly in the same cell; and most aptly a tissue stem cell (Knudson, 1992). The work of these remarkable tissue cell biologists—undertaken at the median of stem cell biology history—set the paradigms for current and future research to discover the biological and evolutionary significance of stem cell genetic fidelity mechanisms. In “Stem Cell Genetic Fidelity,” Lark provides a personal account of the key experimental observation that initiated the field of stem cell genetic fidelity research (Lark et al., 1966). As will be noted in reading the Research Topic, several of the contributed articles are descendants of his seminal contribution.

Here, I join Lark with a somewhat personal account of my own that further builds the context for this Frontiers Research Topic. In the fall 1978 as a new student of cancer research, like many before and after, I set myself on a path to “find a cure for cancer.” However, I was more intrigued with mastering the new DNA maps of transforming viral genomes than the changes that they induced in cells. My studies occurred in the last days before the emergence of the concept that cancer was caused by mutations in cellular genes that resembled the oncogenes carried by tumor-forming viruses. Of course, the tenet that gene mutation was an essential aspect of the carcinogenic mechanism was well established long before the first human cancer gene was identified. So, years before I was drawn to cancer research, many cancer scientists were thinking about how carcinogenic mutations arose. However, there was only one who was asking why there were not *more* carcinogenic mutations, and correspondingly a *higher* incidence of human cancers. John Cairns.

In his 1975 report (Cairns, 1975) on the natural history of human cancers, Cairns introduced the fundamental idea of tissue stem cell genetic fidelity and set in motion the essential hypothesis that would trouble the thoughts of cancer scientists and a parallel universe of stem cell biologists for years to come. His proposal that mammalian tissue stem cells must have a unique mechanism to lower their rate of carcinogenic mutation continues to be a disruptive idea in both stem cell biology and cancer biology.

The blade of Cairns' hypothesis has two sharp edges. First, the “immortal strand hypothesis” cuts through apparent tissue cells and attributes the cell of origin for cancer to tissue stem cells, which are generally physically elusive. Second, the proposed molecular basis for the hypothesis, non-random sister chromatid segregation, slices through geneticists' essential Mendelian laws of mitotic chromosome segregation. Cairns proposed that *asymmetrically* cycling tissue stem cells ignored Mendel's previously immutable laws of random assortment and independent segregation. By non-random co-segregation of the complement of sister chromatids with the older template DNA strands, Cairns saw that asymmetrically cycling tissue stem cells could avoid DNA replication errors, which he predicted to be the most frequent source of carcinogenic mutations.

Cairns' immortal strand hypothesis has met with consternation, skepticism, and outright ridicule. It certainly meets the criteria for a bold, new disruptive idea. Though I had no interest in reading Cairns' paper when it was assigned to my undergraduate Biochemistry course before he arrived as a guest lecturer in the spring of 1978 at Harvard College, in later years I would develop the first theoretical estimate of the extent to which tissue stem cells might lower their mutation rate compared to their differentiating progeny cells (Sherley, 2006); and I would become driven to pursue and promote research to discover the responsible molecular mechanisms. It is my hope that “Stem Cell Genetic Fidelity” will in some measure contribute to this purpose.

## Conflict of interest statement

The author declares that the research was conducted in the absence of any commercial or financial relationships that could be construed as a potential conflict of interest.
